# Patient on Multiple Hormone Replacement Therapy for Hip Arthroplasty: How to Omit Noise and Be Focused?

**DOI:** 10.7759/cureus.27904

**Published:** 2022-08-11

**Authors:** Gautham Patel, Shubhkarman Kahlon, Venkata Ganesh

**Affiliations:** 1 Department of Anaesthesia and Intensive Care, Post Graduate Institute of Medical Education and Research, Chandigarh, IND

**Keywords:** combined spinal-epidural, stress dose of steroids, avascular necrosis (avn), primary adrenal insufficiency, cushing’s disease, total joint arthroplasty

## Abstract

Total hip arthroplasty (THA) surgery is usually performed in patients with trauma or old-aged osteoarthritis. There has been a recent increase in younger patients presenting with avascular necrosis (AVN) of the hip requiring replacement arthroplasty. Despite being from a younger age group, these patients may present with multiple comorbidities. We describe one such case of Cushing’s syndrome with AVN in a young patient with primary adrenal insufficiency, secondary hypothyroidism, and secondary hypogonadism on replacement therapy status post-transsphenoidal pituitary surgery and bilateral adrenalectomy, currently posted for total hip replacement (THR) surgery.

## Introduction

Total hip arthroplasty (THA) surgery is usually performed in patients aged between 60 and 80 years following trauma or osteoarthritis. In recent years, several younger adults are presenting with osteonecrosis of the hip, of which avascular necrosis (AVN) accounts for approximately 70% [1,2]. Although the origin of AVN is multifactorial, major predisposing conditions include alcoholism, smoking, corticosteroid therapy, and early hypertension among nontraumatic cases. Both general and regional anesthesia can be used, but combined spinal-epidural (CSE) is preferred as it has multiple benefits in terms of better postoperative analgesia, lesser blood loss, low incidence of DVT, etc. All of these help to provide better perioperative outcomes with early weight-bearing and shorter hospital stays [3]. In Cushing’s syndrome, AVN of the femoral head can rarely be seen as a presenting feature due to endogenous hypercortisolism, but it most likely occurs as a complication of cortisol therapy. We describe one such case of Cushing’s syndrome with AVN in a young female who presented for THA.

## Case presentation

**Figure 1:**
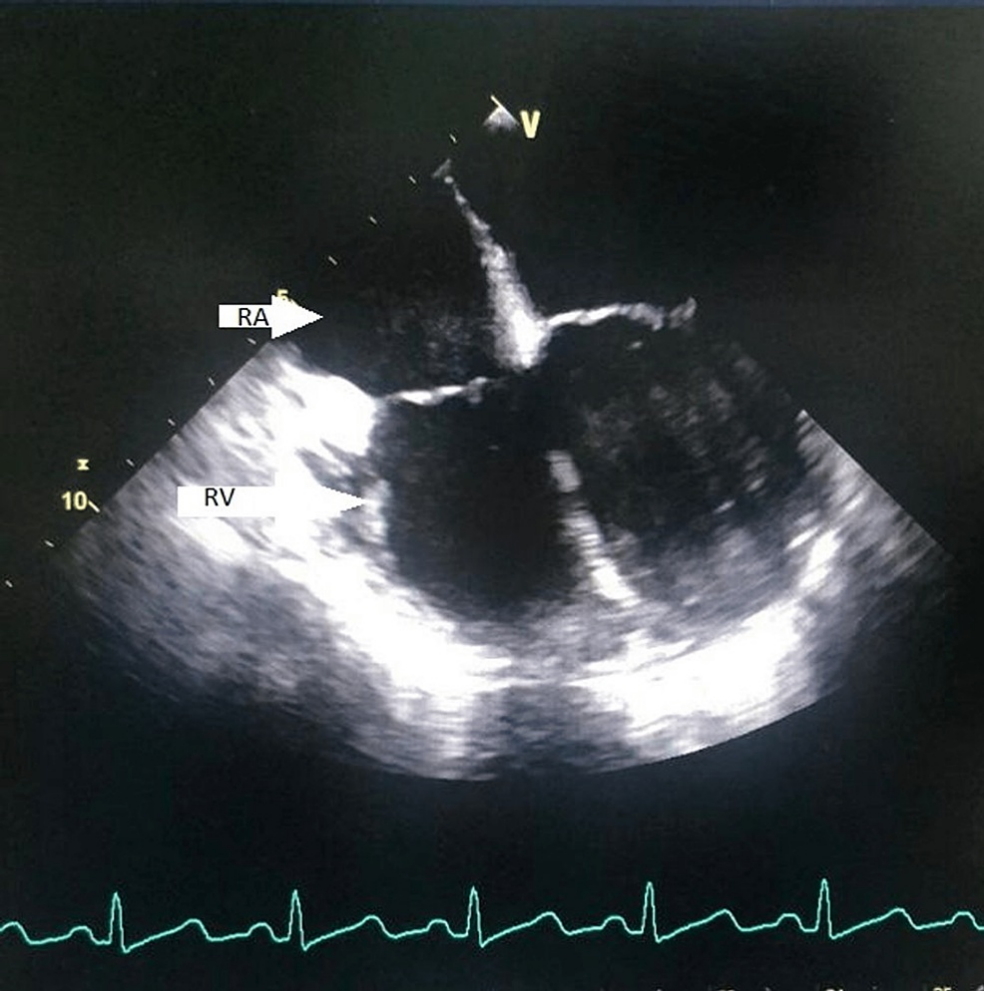
TEE showing mild RV/RA dilatation (arrows) TEE: transesophageal echocardiography; RV: right ventricle; RA: right atrium

A 29-year-old female patient with ACTH-dependent Cushing’s syndrome with localized microadenoma of the pituitary underwent transsphenoidal surgery (TSS) in 2005 as the patient was responding to medical therapy under general anesthesia. Ten months after the TSS, after the ketoconazole trial for adrenal adenoma, she was subjected to bilateral adrenalectomy and was started on hormone replacement therapy with oral prednisone, fludrocortisone, and levothyroxine (LT4). Within one year of corticosteroid therapy, the patient developed bilateral AVN for which bilateral core decompression of the hip was performed. However, this gave only mild symptomatic relief, necessitating a left-sided total hip replacement (THR) under spinal and epidural anesthesia the following year in 2007. However, the perioperative course was complicated by desaturation, and 2D ECHO showed mild right ventricular (RV) enlargement (Figure 1), although CT pulmonary angiogram was negative. The patient was treated with a presumptive diagnosis of pulmonary thromboembolism (PTE) and managed conservatively in the ICU with supplemental oxygen and therapeutic heparin. The patient was discharged after 10 days of ICU stay.

**Figure 2:**
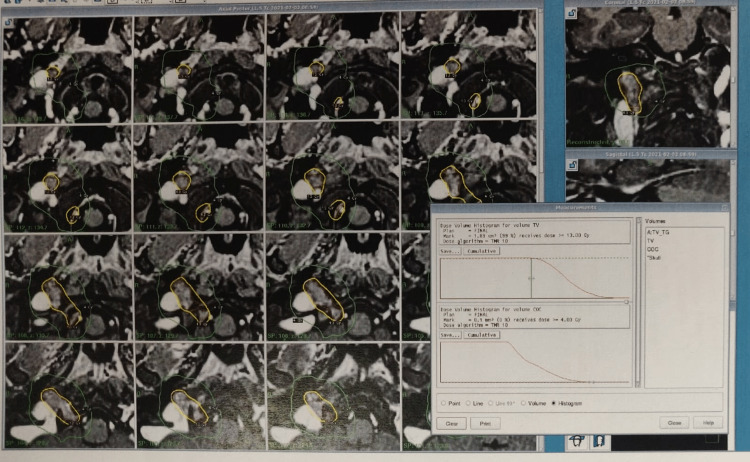
Gamma knife snapshot showing right ICA thrombosis ICA: internal carotid artery

The patient developed Nelson’s syndrome as a complication of bilateral adrenalectomy later despite transsphenoidal resection of pituitary adenoma and had to undergo a redo TSS in 2011. During this surgery, she suffered massive blood loss from an injury to the right meningohypophyseal branch of the internal carotid artery (ICA). She was resuscitated with a massive blood transfusion. Postoperatively, she also underwent a percutaneous stent placement across a saccular aneurysm in the cavernous segment of the right ICA. On postoperative day 7, angiography showed thrombosis of the right ICA for which she underwent gamma knife surgery with monitored anesthesia care (MAC) (Figure 2). Following that, the patient was discharged on oral hydrocortisone 10 mg, fludrocortisone 50 mg, and levothyroxine 125 mcg.

**Figure 3:**
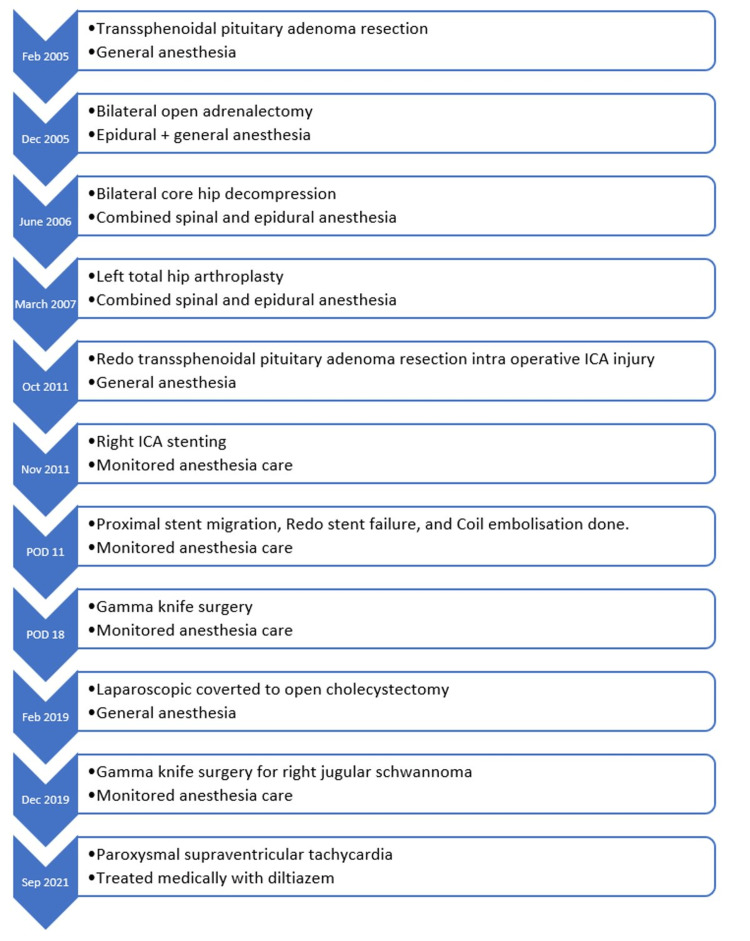
Flow diagram showing the sequence of events prior to admission

In November 2019, the patient developed bulbar palsy, which was treated conservatively. She also developed a right jugular schwannoma, and a gamma knife surgery was performed under MAC for the same in January 2019. In September 2021, the patient developed paroxysmal supraventricular tachycardia (PSVT), which was medically managed, and she was discharged on oral diltiazem (Figure 3).

**Figure 4:**
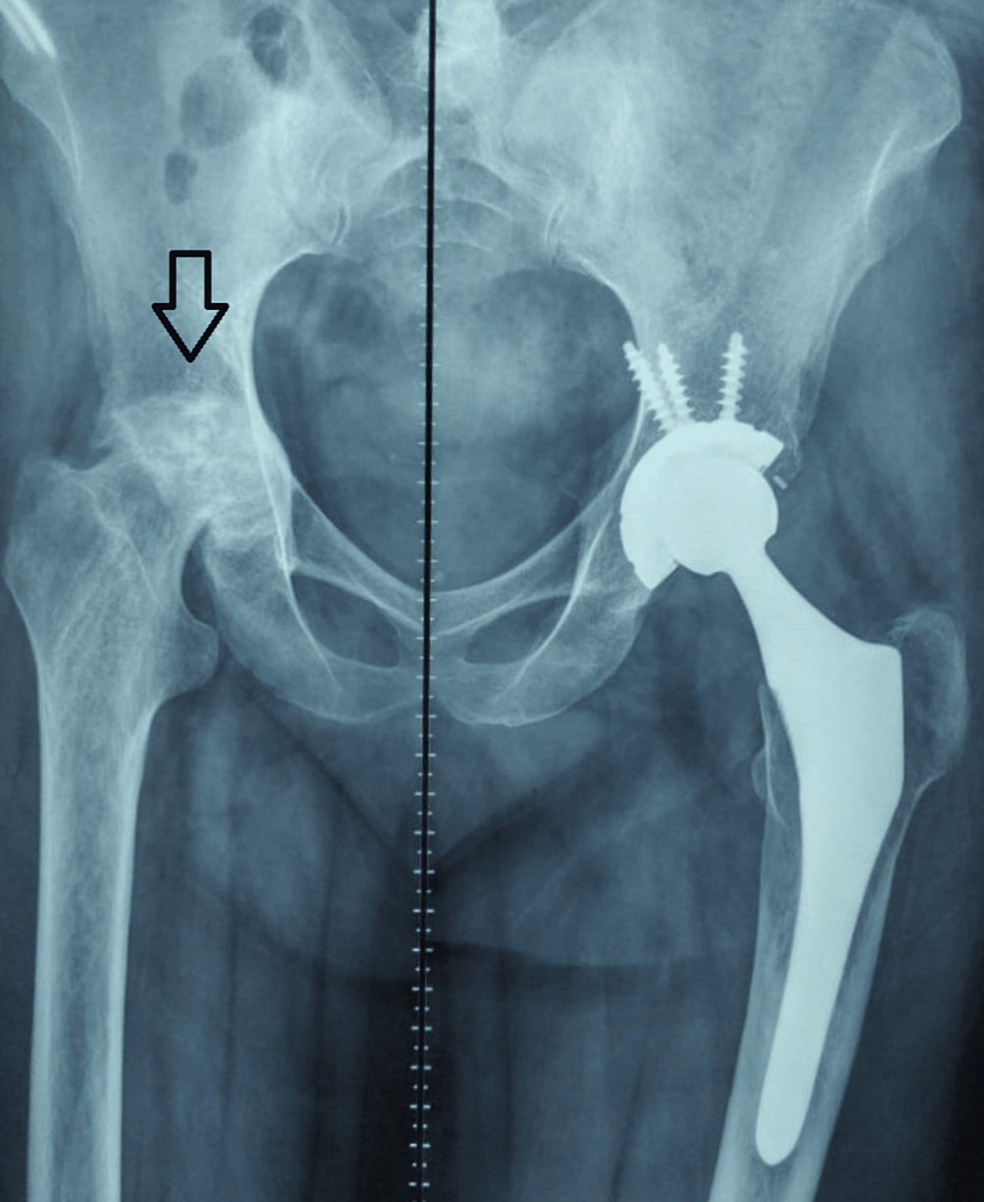
Preoperative X-ray showing right-sided AVN (arrow) AVN: avascular necrosis

In the present admission, the patient was posted for right-sided THR in view of AVN with primary adrenal insufficiency, secondary hypothyroidism, and secondary hypogonadism on replacement therapy (Figure 4).

**Figure 5:**
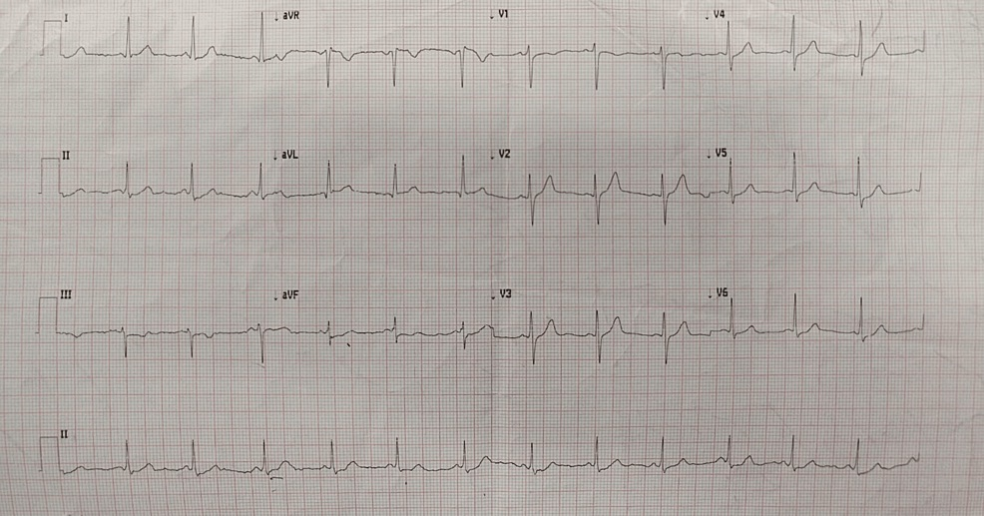
Preoperative ECG

The preoperative anesthetic evaluation revealed a conscious, cooperative, and oriented patient with stable vitals. ECG showed ST depression in lead III, aVF, V5, and V6 for which diltiazem was withheld transiently for stress myocardial perfusion scintigraphy (Figure 5). The stress test showed preserved contractility in all walls of the LV, with a post-stress LVEF of 65%. Preoperatively, diltiazem was restarted and continued. Serum cortisol levels and other blood investigations were within normal limits (Table 1), so in addition to her usual dose of oral steroids, an additional IV stress dose of hydrocortisone was planned.

**Table 1:** Preoperative laboratory investigations

Laboratory parameters	Patient values	Reference values
Hemoglobin	10 g/dL	12-16 g/dL
Total leucocyte count	7.3 × 10^9^/L	4-11 × 10^9^/L
Platelets	149 × 10^9^/L	150-400 × 10^9^/L
Sodium	135 mmol/L	135-145 mmol/L
Potassium	3.54 mmol/L	3.5-5 nmol/L
Urea	20.6 mg/dL	20-40 mg/dL
Creatinine	0.69 mg/dL	0.7-1.1 mg/dL
Cortisol	497 nmol/L	150-550 nmol/L
T3	1.18 ng/mL	0.8-1.8 nm/mL
T4	9.91 mcg/dL	5-12 mcg/dL
TSH	0.005 mcgIU/mL	0.35-5 mcgIU/mL
Free T4	1.61 ng/dL	0.3-2.3 ng/dL

Written and informed consent for central neuraxial anesthesia was taken. After adequate fasting, she was taken up as the first case in the morning after making sure that the patient had taken the morning dose of levothyroxine and the usual dose of steroid. Two wide-bore cannulas were secured, and the preinduction arterial catheter was placed in the left radial artery. Injection hydrocortisone 100 mg bolus was administered intravenously, and an infusion of 4 mg/hour was started, which was continued postoperatively for 24 hours as per institution protocol. A combined spinal epidural block was the anesthetic of choice; given the patient’s previous history of thromboembolic disease, CSE imparts a lower risk of deep vein thrombosis and pulmonary embolism. CSE was performed with a subarachnoid injection of 10 mg of 0.5% hyperbaric bupivacaine with 20 mcg of fentanyl. Once the T10 dermatome was anesthetized, the patient was positioned in the left lateral position for the surgery. Epidural was initiated after 90 minutes of the spinal block with isobaric bupivacaine 0.5% bolus at a rate of 6-8 mL/ hour. Surgery was completed in 2.5 hours, without any significant hypotension. To take care of postoperative pain, 0.125% bupivacaine epidural infusion was started at 5 mL/hour through continuous infusion and was continued for 48 hours. Thromboprophylaxis was initiated the next day, and the epidural catheter was removed as per the American Society of Regional Anesthesia (ASRA) guidelines concerning LMWH dosing. The patient remained stable postoperatively and was discharged on POD 7.

## Discussion

In patients with a long and complex history of multiple comorbidities, it becomes important to eliminate the noise and formulate an approach that is focused and caters to the present needs of the patient. ACTH-dependent Cushing’s disease is usually caused by pituitary adenomas. The treatment of choice is selective pituitary resection including medical management, radiation therapy, and bilateral adrenalectomy [4]. Avascular necrosis is seldom seen as a part of initial case presentation due to endogenous hypercortisolism, but usually, AVN is a complication of prolonged glucocorticoid therapy [5,6]. Patients on chronic steroid therapy may develop adrenal insufficiency because of surgical stress that may lead to a full-blown adrenal crisis in the perioperative period. The adrenal crisis remains a dreaded sequela of bilateral adrenalectomy, and the incidence of morbidity and mortality can be as high as 64% and 22%, respectively [7]. Hence, it is empirical to ensure normal preoperative serum cortisol and thyroxine levels. Perioperatively, patients with adrenal crisis present with an unexplained shock that is refractory to vasopressors and fluids associated with hyponatremia, hyperkalemia, hypoglycemia, low or low normal ACTH level in secondary adrenal insufficiency and high or high normal ACTH level in primary adrenal insufficiency, hypercalcemia, prerenal failure with elevated creatinine level, and low aldosterone. The definitive treatment of adrenal crisis is the administration of glucocorticoids, specifically hydrocortisone. IV hydrocortisone (100 mg) should be administered, followed by 100 mg IV every 6-8 hours. Since dehydration and hypovolemia are common precipitating factors, rehydration of the patient with 0.9% normal saline is essential. The correction of hypoglycemia avoids rapid correction of hyponatremia of more than 6-8 mEq in the first 24 hours. The tapering of steroids should be gradual after there is a clinical improvement [8].

Hence, weighing the risk of adrenal crisis to unnecessary steroid supplementation, stress dosing is clinically recommended [9]. Hydrocortisone is usually the drug of choice. In known hypothyroidism, if patients develop adrenal insufficiency, it should be made sure the latter is treated before the former. This is because the administration of thyroxine may enhance cortisol clearance and increase basal metabolic rate, which may lead to increased requirement of cortisol. Both of these may precipitate an adrenal crisis [10]. Also, since these patients have 10 times higher odds of atrial fibrillation and PTE, it is empirical for continuous cardiac monitoring [11].

## Conclusions

To conclude, in these patients, a holistic approach with interdepartmental coordination, especially with an endocrinologist, is important. Meticulous preoperative evaluation, optimization, and consideration of all possible complications are the cornerstone of management. Perioperatively, an anesthesiologist should have a keen eye to look for volume overload, hyperglycemia, hypokalemia, difficult airway, and ventilation. As hip replacement surgeries are now on the rise in young patients with multiple comorbidities, it is empirical for anesthetists to learn, practice, and adapt to the new trend.
